# Complexes of Soluble Dietary Fiber and Polyphenols from Lotus Root Regulate High-Fat Diet-Induced Hyperlipidemia in Mice

**DOI:** 10.3390/antiox13040466

**Published:** 2024-04-16

**Authors:** Zhan Zheng, Weilan Gao, Zhenzhou Zhu, Shuyi Li, Xueling Chen, Giancarlo Cravotto, Yong Sui, Lei Zhou

**Affiliations:** 1 National R&D Center for Se-Rich Agricultural Products Processing Technology, School of Modern Industry for Selenium Science and Engineering, Wuhan Polytechnic University, Wuhan 430023, China; 17671055616@163.com (Z.Z.); weilangao@126.com (W.G.); 2 Key Laboratory of Agro-Products Cold Chain Logistics, Ministry of Agriculture and Rural Affairs, Institute of Agro-Products Processing and Nuclear-Agricultural Technology, Hubei Academy of Agricultural Science, Wuhan 430064, China; chenxueling13@hbaas.com (X.C.); suiyong_1231@hbaas.com (Y.S.); zhouleidjj@163.com (L.Z.); 3 Department of Drug Science and Technology, University of Turin, 10125 Turin, Italy; giancarlo.cravotto@unito.it

**Keywords:** soluble dietary fiber, polyphenols, complexes, lipid metabolism, gut microbiota, hyperlipidemia

## Abstract

In this paper, complexes of soluble dietary fiber (SDF) and polyphenols (PPs) isolated from lotus roots were prepared (SDF-PPs), as well as physical mixtures (SDF&PPs), which were given to high-fat-diet (HFD)-fed mice. The results demonstrated that SDF-PPs improve lipid levels and reverse liver injury in hyperlipidemic mice. Western blotting and real-time quantitative Polymerase Chain Reaction (RT-qPCR) results showed that SDF-PPs regulated liver lipids by increasing the phosphorylation of Adenine monophosphate activated protein kinase (AMPK), up-regulating the expression of Carnitine palmitoyltransferase1 (CPT1), and down-regulating the expression of Fatty acid synthase (FAS) and 3-hydroxy-3-methyl glutaryl coenzyme A (HMG-CoA), as well as the transcription factor sterol-regulatory element binding protein (SPEBP-1) and its downstream liposynthesis genes. Additionally, the intervention of SDF-PPs could modulate the composition of intestinal gut microbes, inducing an increase in *Lachnospiraceae* and a decrease in *Desulfovibrionaceae* and *Prevotellaceae* in high-fat-diet-fed mice. Thus, the research provides a theoretical basis for the application of lotus root active ingredients in functional foods and ingredients.

## 1. Introduction

Numerous disorders collectively known as hyperlipidemia are characterized by increased levels of total cholesterol (TC), triglycerides (TGs), and low-density lipoprotein cholesterol (LDL-C) and decreased levels of high-density lipoprotein cholesterol (HDL-C) [1], and liver damage and intestinal imbalance are complications [2,3]. The nutritional structure of the population has altered significantly due to the fast development of the economy. High fat and protein consumption, along with a lack of activity, results in younger people developing metabolic illnesses, such as obesity, diabetes, and hyperlipidemia. Hyperlipidemia poses a serious concern for human health, since it can hasten the initiation and development of atherosclerosis and turn into a major risk factor for heart and brain conditions [4]. Statins and fibrates are currently the most often used treatment medications for hyperlipidemia. Despite their amazing potency, these two classes of medicines have a single mode of action. Statins have also been linked to side effects, such as liver damage, rhabdomyolysis, and the development of new tumors, as well as a high rebound rate following withdrawal [1,5]. As a result, many functional foods with hypolipidemic effects have received a lot of interest, and the mechanism of action of these active ingredients has become a research hotspot.

Lotus root belongs to the rhizome of the lotus plant of *Nymphaeaceae* and contains a great number of biological bioactive components, such as phenolics and dietary fiber. The main phenolic substances in lotus root are pyrogallic gallic acid, gallic acid, caffeic acid, catechol, catechin, and epicatechin, which is consistent with the antioxidant, anti-inflammatory, and hypoglycemic activities of lotus root polyphenols that have been reported [6]. On the other hand, the content of dietary fiber in lotus root is reported to be 35.95%, while the content of soluble dietary fiber accounts for 9.20% [7]. Dietary fiber is crucial for regulating microbial growth and intestinal peristalsis. It has been demonstrated that non-metric bonds may connect phenolics and dietary fiber into complexes, increasing the stability of polyphenols [8]. Dietary fiber–polyphenols complexes have important bioactive functions in the body, including the regulation of gut microbiota and reducing inflammation [9]. Previous studies from our lab have found that lotus root soluble dietary fiber (SDF) and polyphenols (PPs) can form complexes through interaction, and the in vitro antioxidant and lipid-lowering activities of the two showed a synergistic effect after the interaction, but there is a lack of research on the lipid-lowering effect in vivo and the mechanism of action of the complexes.

Because adipocyte proliferation and hypertrophy cause adipose tissue to accumulate in the form of obesity, adipogenesis is a major factor in the emergence of obesity and an important target for obesity prevention. The fundamental regulator of cellular energy, Adenine monophosphate activated protein kinase (AMPK), is essential for regulating fat production and managing lipid metabolism [10]. Acetyl CoA carboxylase-1 (ACC1), fatty acid synthase (FAS), and sterol-regulatory element binding protein (SREBP-1) expression are all inhibited by AMPK after it has been activated by phosphorylation, which lowers the synthesis of fat and cholesterol [11]. Han discovered that Sawtooth leaf hot water extract reduced the accumulation of fat in mice by activating AMPK, inhibiting the AKT/mTOR pathway, and reducing the expression of PPARγ, C/EBPα, and SREBP-1 genes [12]. Li discovered that *L. fermentum* HFY06 and arabinoxylan worked together to regulate lipid metabolism in vitro by activating the AMPK pathway; up-regulating the mRNA expression of CPT-1, PPAR-α, CYP7A1, and HSL; and alleviating hyperlipidemia [13]. Activating the AMPK pathway has been found to reduce dyslipidemia and the hepatic fat accumulation in mice brought on by a high-fat diet; however, there are still few findings on how lotus root extract promotes AMPK phosphorylation.

Recent research has demonstrated that, by controlling energy balance, the gut microbiota plays a critical role in the pathogenesis of obesity. The gut microbiota, which comprises more than 100 trillion microorganisms, is found in the human intestine. Obesity, hyperlipidemia, diabetes, and nonalcoholic fatty liver disease (NAFLD) are only a few of the metabolic illnesses that are linked to disturbed gut microbiota balance [14]. Diet is one of the most crucial variables in establishing gut homeostasis, and it naturally interacts with gut bacteria [15]. According to a study, cranberry extract improved the metabolic syndrome brought on by a high-fat diet by increasing the number of *Akkermansia* bacteria in the intestine [16]; Yu discovered that increasing the therapeutic dose of inulin to treat hyperlipidemia led to an increase in the number of *Bifidobacteria* in the digestive tract of hyperlipidemic mice [17]. Therefore, altering the gut’s microbial composition would help to control lipid metabolism and improve illness outcomes. The ameliorative impact of lotus root complexes on the gut flora of hyperlipidemic mice, however, has not been documented in the literature.

In this study, we aimed to clarify how soluble dietary fiber and polyphenols complexes (SDF-PPs) may reduce blood lipid levels. Fresh lotus root was used as a raw material for making the lotus root soluble dietary fiber and polyphenols complexes. Mice were given high-fat feed (HFD) to induce hyperlipidemia, and changes in blood lipids, inflammatory factor levels, and oxidative responses were observed after the intervention of SDF-PPs, and the effects of SDF-PPs on important genes for lipid metabolism and the AMPK phosphorylation pathway in mice were examined by Western blotting and real-time quantitative fluorescence PCR. Additionally, through gut microbial diversity studies, the possible association between lipid-lowering effects and gut bacteria is examined. This study will provide a new possibility for the dietary treatment of hyperlipidemia.

## 2. Materials and Methods 

### 2.1. Materials

The lotus roots used in this study were provided by the Hubei Provincial Academy of Agricultural Sciences. First, the polyphenols (PPs) from lotus roots were prepared according to the method described by Tsuruta with some modifications [18]. Subsequently, the soluble dietary fiber (SDF) was extracted by MES-TRIS buffer. In short, this process is a continuous enzymatic hydrolysis with α-amylase, protease, and saccharifying enzymes to remove starch and protein from the sample, and after the enzymatic hydrolysis, centrifugation separates the soluble dietary fiber. The lotus root soluble dietary fiber–polyphenols complexes were prepared according to the method described in the literature by dissolving SDF (480 mg) in 240 mL of purified water and PPs (120 mg) in 120 mL of purified water and then adding the PPs solution to the SDF solution (*v*:*v*, 1:2) and stirring it for 20 min. Finally, the product was transferred to a pretreated dialysis bag (Mw cutoff: 1000 Da, Beijing Solarbio Technology Co., Ltd., Beijing, China) until the adsorption of polyphenols was equilibrated and there was no change in the phenolic content in the fluid outside the dialysis bag [19]. The dialysis solution was collected and lyophilized to obtain soluble dietary fiber–polyphenols complexes (SDF-PPs), which contained 37.50% polyphenols. Physical mixtures (SDF&PPs) were prepared by mixing SDF and PPs according to the ratio of soluble dietary fiber to polyphenols in the complexes. SDF-PPs were obtained via the non-covalent interaction of SDF and PPs, whereas the SDF&PPs were simply a mixture of the two substances. In this study, the basal feed (the diet provided 3.044 kcal/g) and the high-fat and high-sugar feed (the diet provided 3.941 kcal/g) were purchased from Wuhan Wanqian Jiaxing Biotechnology Co., Ltd., Wuhan, China.

### 2.2. Animal Experiment Design

The experiment complied with the National Guidelines for Laboratory Animal Welfare (MOST of PR China, 2006), and the experimental protocol was approved by the Hubei Provincial Center for Disease Control and Prevention (SYXK(e) 2022-0065). The mice were kept in a controlled environment with a day/night cycle of 12 h, a temperature of 22 ± 2 °C, and a relative humidity of 60 ± 5%. Eighty-four mice were habitually fed with a normal control diet and water for one week in the laboratory environment. The mice were randomly divided into seven groups (n = 12): a normal control group (NC), a model control group (MC), a positive control group (simvastatin 10 mg/kg, PC), a soluble dietary fiber group (250 mg/kg, SDF), a polyphenols group (150 mg/kg, PPs), a physical mixing group (250:150 ratio blend, SDF&PPs), and a composite group (400 mg/kg, SDF-PPs), and the details of the groupings are shown in Figure S1. The NC group was fed a normal diet, and the rest of the groups were subjected to hyperlipidemia modeling because C57BL/6J is sensitive to feed with a high calorie content and can be easily modeled successfully by administering a high fat feed. So, high-fat-diet feeding (HFD, consisting of 66.5% normal diet, 10% lard, 20% sucrose, 2.5% cholesterol, and 1% sodium cholate) was selected. It is noteworthy that the hyperlipidemia model was constructed in the MC group along with a gavage intervention in the other groups, and the PC group was gavaged with 10 mg/kg simvastatin; the simvastatin dose was obtained based on the equivalent dose ratio converted by body surface area between humans and animals (70 kg, 80 mg/kg/d). The intervention groups were gavaged with SDF, PPs, SDF&PPs, and SDF-PPs. All experimental groups were gavaged at the same time, at noon each day. Body weight and food intake were tracked weekly during the seven-week experimental period. The mice were subjected to a 12 h fast and water restriction one day prior to the beginning of the seventh week. Blood samples from the mice’s orbital venous plexus were collected using glass capillaries, and the levels of TC, TGs, and LDL-C were determined and compared to those from the control group. Notably, significantly elevated serum levels of TC, TGs, and LDL-C in the modeling group of mice were considered to be the criteria for the success of the hyperlipidemia model [20]. All mice were fasted with food and water for 12 h after the last gavage. Blood was taken from the eye sockets, cervical dislocation was performed for execution, and fresh organs were quickly removed and weighed to calculate the organ indices. A quantity of 4% paraformaldehyde was used to fix one piece of the liver. The cecum’s contents were removed and put in a 2 mL freezer tube with a screw-top lid. In order to quickly freeze the organs and cecum contents, liquid nitrogen was used first, followed by backup storage at −80 °C.

### 2.3. Hematological Analysis

The levels of serum triglycerides (TGs); total cholesterol (TC); high-density lipoprotein (HDL-C); low-density lipoprotein (LDL-C); and alkaline phosphatase (ALP), aspartate transaminase (AST), and alanine transaminase (ALT) enzymes were measured using an automated biochemical analyzer (Chemray 240, Rayto Life and Analytical Sciences Co., Ltd., Shenzhen, China).

### 2.4. Determination of Enzyme Activity and Pro-Inflammatory Cytokines in Liver

Superoxide dismutase (SOD), glutathione peroxidase (GSH-Px), malondialdehyde (MDA), advanced glycation end products (AGEs), and total antioxidant capacity (TAOC) in the liver homogenate were detected by commercial kits and following the manufacturers’ instructions. Determination kits were purchased from Nanjing Jiancheng Bioengineering Institute (Nanjing, China). The protein expression levels of interleukin-1β (IL-1β), interleukin-6 (IL-6), tumor necrosis factor-α (TNF-α), and transforming growth factor β (TGF-β) in the liver homogenate were determined using ELISA. ELISA kits were purchased from Jiangsu Enzyme Immunoassay Industry Co., LTD (Yancheng, China).

### 2.5. Histopathology Analysis

Paraformaldehyde-fixed liver tissues were obtained, dehydrated with gradient ethanol, rinsed with xylene, embedded in paraffin, sectioned (4 μm), stained with hematoxylin–eosin, examined under a microscope, and photographed for documentation.

### 2.6. AMPK Pathway-Related Protein and Gene Expression Assays

#### 2.6.1. Western Blot Analysis

Liver tissue was washed 2–3 times using cold Tris-buffered salt solution (TBS) to remove blood stains. After weighing 20 mg of liver tissue and adding 200 μL of lysis solution (P0013B, Biyuntian, Shanghai, China), protease inhibitor (P1081, Biyuntian, Shanghai, China) was added before the lysis solution was used, whereupon it was placed in 2 mL milling tubes, steel beads were added, and it was milled using a milling instrument until it was sufficiently lysed. 20 mg of liver tissue was weighed and added to 200 µL of lysis solution (P0013B, Biyuntian, Shanghai, China), then placed in a 2 m grinding tube with steel beads and ground with a grinder until fully lysed. After sufficient grinding, centrifugation was performed at 12,000 rpm at 4 °C for 10 min and the supernatant was collected, which was the total protein solution. Then, white quantification was performed strictly according to the BCA protein quantification assay kit process (P0012, Biyuntian, Shanghai, China). Finally, the protein concentration of each sample was adjusted to the same concentration using PBS. Whole proteins (0.20 μg) were electrophoresed by 10% SDS-PAGE at 75 V for the concentration gel and 100 V for the separation gel, and electrophoresis was stopped after the bromophenol blue was almost completely consumed. The membrane was then transferred to a PVDF membrane and sealed with 5% skimmed milk for two hours at room temperature. Protein samples were then treated with a 1:1000-fold dilution of primary antibody (CPT1, p-AMPK, AMPK, HMG-CoA, FAS, GAPDH; Proteintech Group, Inc., Wuhan, China) at 4 °C overnight. The secondary antibody was diluted with TBST (1:5000, HRP-Goat anti Rabbit, 111-035-003, 1:5000, jackson; HRP-Goat anti mouse, SA00001-1, 1:5000; Proteintech Group, Inc.,Wuhan, China), incubated for 30 min at room temperature, and then washed three times with TBST on a decolorizing shaker at room temperature. The protein side of the PVDF membrane was placed face up in full contact with the ELC mixture, and then the PVDF membrane was placed in an exposure meter (Tanon-4800, Shanghai, China) to measure the optical density of the target bands to determine whether the target protein was present on the membrane.

#### 2.6.2. Real-Time Quantitative Polymerase Chain Reaction Analysis

Fresh liver tissue weighing about 100 mg was taken and 1 mL of Trizol reagent (R401-01, Vazyme, Nanjing, China) was added, followed by homogenization with a homogenizer, and then 200 μL of chloroform was added and mixed, and the mixture was left at room temperature for 5 min, then centrifuged at 12,000 r/min at 4 °C for 15 min. The upper aqueous phase was then taken, isopropanol was added in an equal volume, mixed well, and then the mixture was left at room temperature for 10 min, followed by centrifugation at 12,000 r/min for 10 min at 4 °C, after which the white precipitate was washed with 1 mL of 75% anhydrous ethanol several times. The purity and concentration of RNA were measured using a microspectrophotometer (NanoVue Plus, Haverhill, MA, USA). Following this, a two-step method was used to reverse transcribe RNA into cDNA. In the first step, the gDNA was removed; the reaction system was 4 μL of 4 × gDNA wiper rMix (R223-01, Vazyme, Nanjing, China) and 4 μg of total RNA. The reaction was carried out at 42 °C for 2 min. For the second step of reverse transcription of the RNA, the reaction system was 4 μL of 5 × HiScripII qRT SuperMix II (R233-01, Vazyme, Nanjing, China) and the reaction solution of the first step, and the reaction conditions were as follows: 50 °C, 15 min; 85 °C, 5 s. Real-time fluorescence quantitative PCR was then performed using the primers shown in Table S1. For sample normalization, the housekeeping gene GAPDH was used. To determine the relative expression of the genes, 2^−ΔΔCT^ was used to examine the final data.

### 2.7. Gut Microbiota Analysis

Total genomic DNA was extracted from mouse cecum contents using the TGuide S96 Magnetic Soil/Stool DNA Kit (Tiangen Biotech (Beijing) Co., Ltd., Beijing, China) according to the manufacturer’s instructions. With the primer pair 338 F: 5′-ACTCCTACGGGAGGCAGCA-3′ and 806 R: 5′-GGACTACHVGGGTWTCTAAT-3′, the hypervariable region V 3–V 4 of the bacterial 16S rRNA gene was amplified. On an agarose gel, the PCR results were validated, and they were then purified using an Omega DNA purification kit (Omega Inc., Norcross, GA, USA). The paired ends (2 × 250 bp) were carried out on the Illumina Novaseq 6000 (Tsingke Biotechnology Co., Ltd., Beijing, China) platform using the purified PCR products that had been collected. The qualifying sequences that met the 97% similarity criterion were assigned to a single operational taxonomic unit (OTU). The OTUs and ASVs were taxonomically annotated at a confidence level of 70%. Alpha diversity evaluated the species richness and species diversity of individual samples, and the degree of similarity in species diversity present in different samples was compared by Beta diversity. The abundance and diversity of bacteria were compared using one-way analysis of variance. The differentially abundant taxa were assessed using linear discriminant analysis (LDA) and effect sizing (LEfSe).

### 2.8. Statistical Analysis

All of the data were statistically analyzed using SPSS statistical software (version 1.1), and the outcomes were reported as means ± SDs and displayed using GraphPad Prism (version 8.0.2). One-way ANOVA was used to compare the means of different groups, and multiple comparisons were made if there was a significant difference between the groups. An independent-samples *t*-test was then used to examine significant differences between the two groups. Statistics were considered significant at *p* < 0.05.

## 3. Results

### 3.1. Effects of SDF-PPs Complexes on the Weight Gain, Food Intake, and Organ Indices of HFD Mice

Chronically consuming a lot of fat raises blood triglyceride and cholesterol levels, which contributes to making the host obese and the development of hyperlipidemia. Table 1 displays the effects of dietary intervention on weight increase, food intake, and the organ index. There was no significant difference in the initial body weight of each group. After 7 weeks of dietary intervention, weight gain was 4.93 g in the MC group and 3.00 g in the NC group, which was 64.33% higher in the MC group than in the NC group. The interventions of SDF, PPs, SDF-PPs, and SDF&PPs all reduced the body weight gain caused by the high-fat diet, especially in the SDF-PPs group.

### 3.2. Effect of SDF-PPs Complexes on the Blood Lipids of HFD Mice

**Figure 1:**
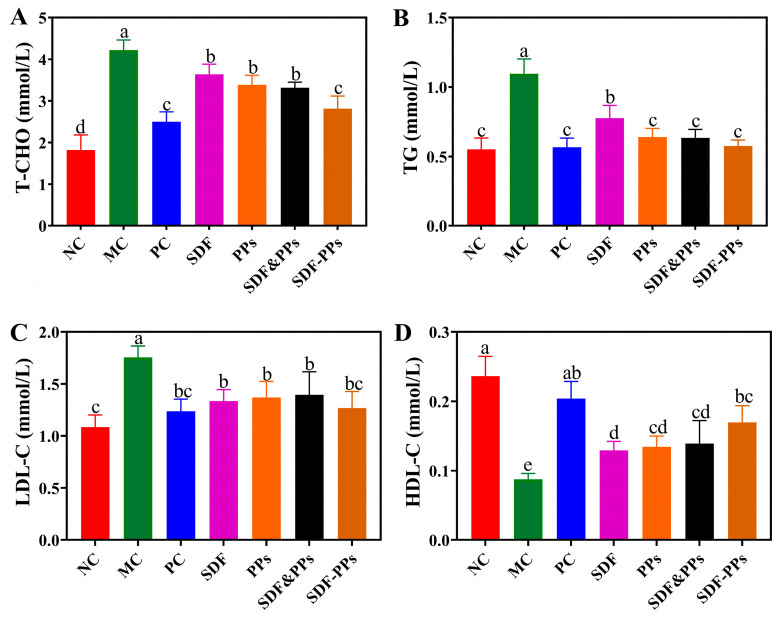
Effect of SDF-PPs complexes on the blood lipids of HFD mice. (**A**) TC. (**B**) TGs. (**C**) LDL-C. (**D**) HDL-C. (a–e) Mean values with different letters in the same figure are significantly different (*p* < 0.05) according to Duncan’s multiple range test. Data are expressed as means ± SDs (n = 6). NC: normal control group; MC: model control group; PC: positive control group (simvastatin 10 mg/kg); SDF: soluble dietary fiber group (250 mg/kg); PPs: polyphenols group (150 mg/kg); SDF&PPs: physical mixing group (250:150 ratio blend); SDF-PPs: composite group (400 mg/kg).

The effects of SDF, PPs, and their complexes on the blood lipids in hyperlipidemic mice are shown in Figure 1A–D. A high-fat diet is frequently to blame for dyslipidemia, as seen by elevated TC, TGs, and LDL-C or decreased HDL-C. The MC group’s TC, TGs, and LDL-C levels increased by 131.87%, 98.18%, and 62.96%, respectively, in contrast to the NC group, and HDL-C declined by 62.50%, proving the mouse hyperlipidemia model’s efficacy. Compared with the MC group, the interventions of SDF, PPs, SDF-PPs, and SDF&PPs all alleviated the dyslipidemia induced by the high-fat diet. According to the results, SDF-PPs were most effective at lowering the mice’s serum levels of TGs and HDL-C, and there was no discernible difference between the other groups’ efforts to lower TGs and LDL-C.

### 3.3. Oxidative Stress Indicators and Inflammatory Factors of HFD Mouse Liver

The antioxidant activity of SDF, PPs, and their complex mixtures on the mouse livers is shown in Table 2. Liver TAOC, SOD, and GSH-Px levels were decreased and MDA and AGEs levels were increased in mice in the MC group compared to the NC group. The SDF-PPs intervention increased TAOC, SOD, and GSH-Px activities while it decreased MDA levels and AGEs contents, whereas SDF, PPs, and SDF&PPs were ineffective in decreasing liver oxidative capacity. Our findings showed that mice fed a high-fat diet had lower levels of the anti-inflammatory factor TGF-β and higher levels of the inflammatory factors IL-6, IL-1β, and TNF-α in their livers. In comparison to the MC group, the levels of IL-6, IL-1β, TNF-, and TGF-β were statistically significant in the SDF, PPs, SDF&PPs, and SDF-PPs groups, and SDF-PPs decreased IL-6, IL-1β, and TNF-α by 50.39%, 55.24%, and 48.88%, respectively, and elevated TGF-β by 113.71%.

### 3.4. Enzyme Activities and Histopathologic Analysis of the HFD Mouse Livers

**Figure 2:**
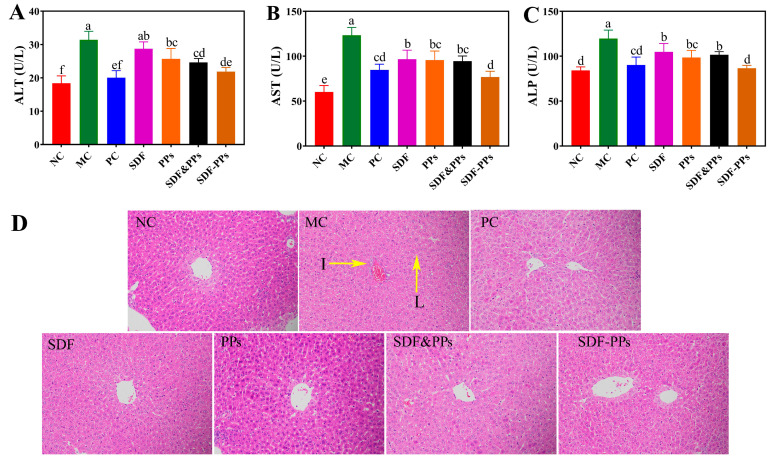
Enzyme activities and histopathologic analysis of the HFD mouse livers. (**A**) ALT. (**B**) AST. (**C**) ALP. (**D**) Histopathologic analysis (×200, Scar bar: 50 μm). “I” indicates inflammatory cell infiltration, and “L” indicates lipid droplet. (a–f) Mean values with different letters in the same figure are significantly different (*p* < 0.05) according to Duncan’s multiple range test. Data are expressed as means ± SDs (n = 6). NC: normal control group; MC: model control group; PC: positive control group (simvastatin 10 mg/kg); SDF: soluble dietary fiber group (250 mg/kg); PPs: polyphenols group (150 mg/kg); SDF&PPs: physical mixing group (250:150 ratio blend); SDF-PPs: composite group (400 mg/kg).

A high-fat diet harms the liver in mice, which causes an increase in serum levels of the enzymes typically used to detect liver injury responses, alanine aminotransferase (AST), alanine aminotransferase (ALT), and alkaline phosphatase (ALP) [21]. While ALT, ALP, and AST activities were considerably decreased by the SDF, PPs, SDF-PPs, and SDF&PPs interventions, SDF-PPs lowered liver enzyme activity levels to the best possible extent, lowering ALT, ALP, and AST activities by 30.31%, 27.74%, and 37.73%, respectively (Figure 2A–C). After 7 weeks of intervention, the influence on the shape of liver cells in the mice was detected (Figure 2D). It was found that the fat cells in the NC group were regularly arranged and that the cells were uniform, while the liver tissues of the MC group showed a large number of fat droplets and the cells were arranged in a disordered manner, in addition to showing obvious inflammatory infiltration. While SDF, PPs, SDF-PPs, and SDF&PPs inhibited the accumulation of fat droplets in hepatocytes and slowed down inflammatory infiltration to a certain extent, the section results of the intervention group were closer to those of the NC group. This is consistent with the results for SDF-PPs with respect to reducing serum lipid levels, which provides strong support for their use as a treatment of dyslipidemia caused by a high-fat diet.

### 3.5. Effects of SDF-PPs Complexes on AMPK Phosphorylation and Lipid Metabolism in HFD Mice

**Figure 3:**
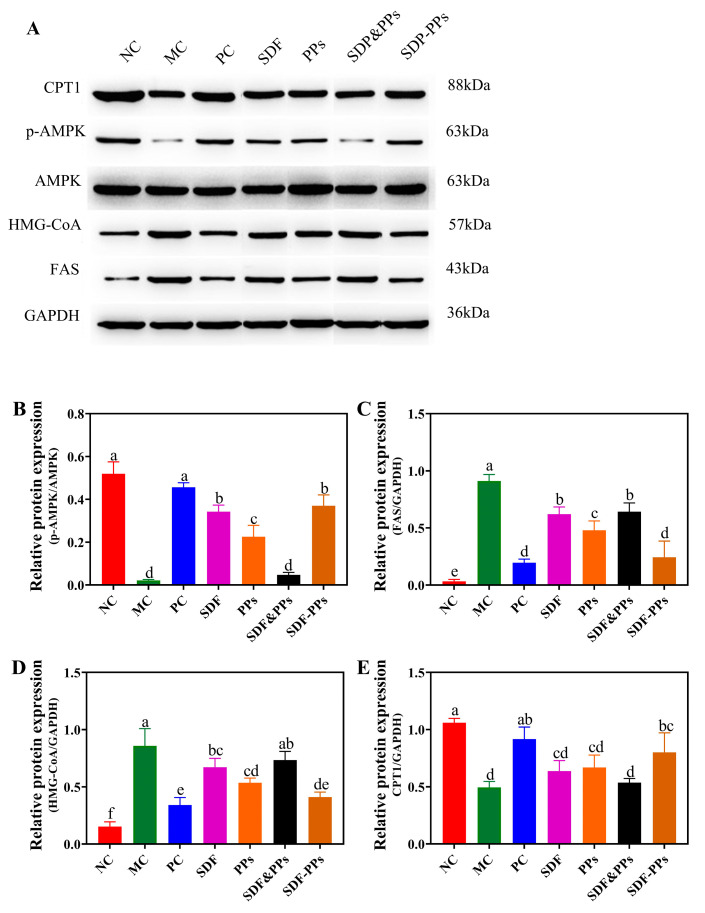

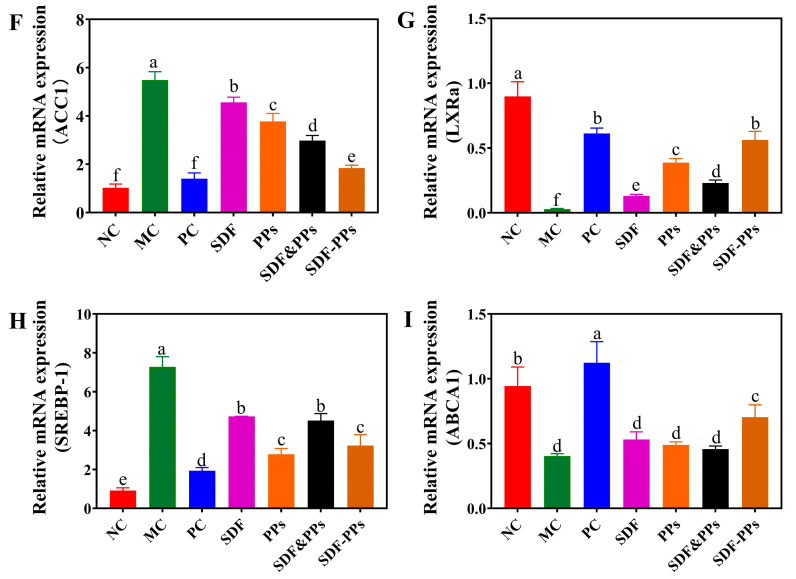
Effects of SDF-PPs complexes on AMPK phosphorylation and lipid metabolism in HFD mice. (**A**) The protein expression levels of p-AMPK, AMPK, HMG-CoA, FAS, and GAPDH in liver tissue were measured by Western blotting. (**B**) p-AMPK/AMPK. (**C**) FAS/GAPDH. (**D**) HMG-CoA/GAPDH. (**E**) CPT1/GAPDH. (**F**–**I**) Expression of the liver metabolic genes SREBP-1, FAS, ACC1, LXRa, and ABCA1. (a–f) Mean values with different letters in the same figure are significantly different (*p* < 0.05) according to Duncan’s multiple range test. Data are expressed as means ± SDs (n = 4). NC: normal control group; MC: model control group; PC: positive control group (simvastatin 10 mg/kg); SDF: soluble dietary fiber group (250 mg/kg); PPs: polyphenols group (150 mg/kg); SDF&PPs: physical mixing group (250:150 ratio blend); SDF-PPs: composite group (400 mg/kg).

We used Western blotting to identify the protein expression of AMPK, p-AMPK, FAS, CPT1, and HMG-CoA in the mice’s livers in order to better understand the potential mechanism of SDF-PPs in controlling lipid metabolism (Figure 3A). As shown in Figure 3B, a high-fat meal considerably reduced the expression of p-AMPK in mice compared to the NC group. SDF, PPs, and SDF-PPs gavage could effectively reverse the reduction in p-AMPK/AMPK, but the improvement effect of SDF&PPs gavage was not immediately apparent. The expression of FAS and HMG-CoA in the proteins was dramatically elevated by the high-fat feed (Figure 3C,D), and after the gavage intervention, both showed an improvement effect; however, the improvement effect of the SDF-PPs group was superior to that of the SDF, PPs, SDF&PPs groups. The high-fat diet also dramatically decreased CPT1 expression when compared to the NC group (Figure 3E), which was significantly restored by the intervention of SDF-PPs (*p* < 0.05). In addition to controlling the activity of lipid metabolism synthases, AMPK also controls the expression of genes associated with lipid synthesis. The expression of the genes SREBP-1, ACC1, LXRa, and ABCA1 associated with mouse liver lipogenesis was examined using real-time quantitative PCR. According to the findings, as shown in Figure 3F–I, gene expression of ACC1 and ABCA1 was significantly elevated in MC, while LXRa and SREBP-1 gene expression was significantly suppressed. The interventions of SDF, PPs, SDF-PPs, and their physical mixtures were found to alleviate the above conditions induced by the HFD to some extent (*p* < 0.05).

### 3.6. Gut Microbiology Analysis

**Figure 4:**
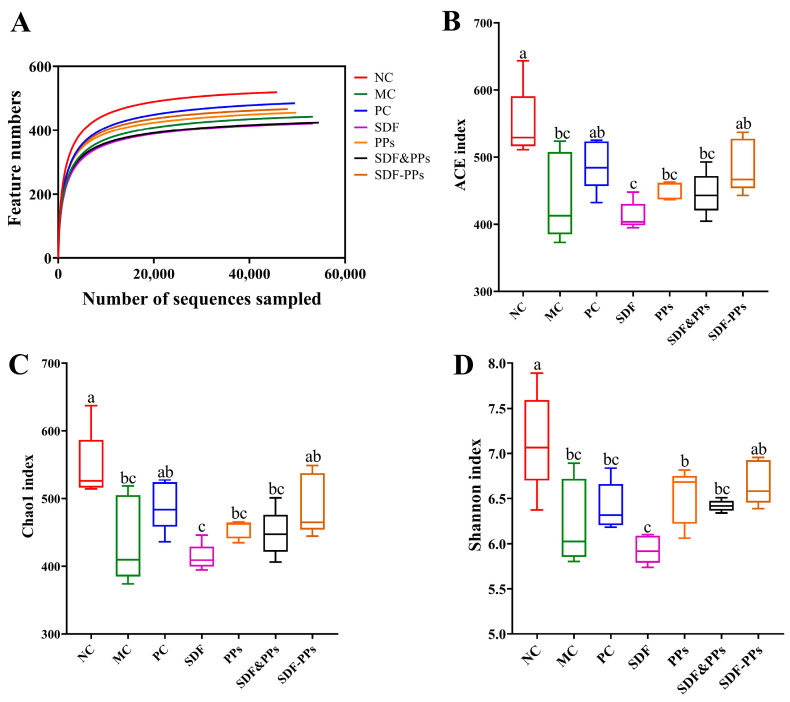

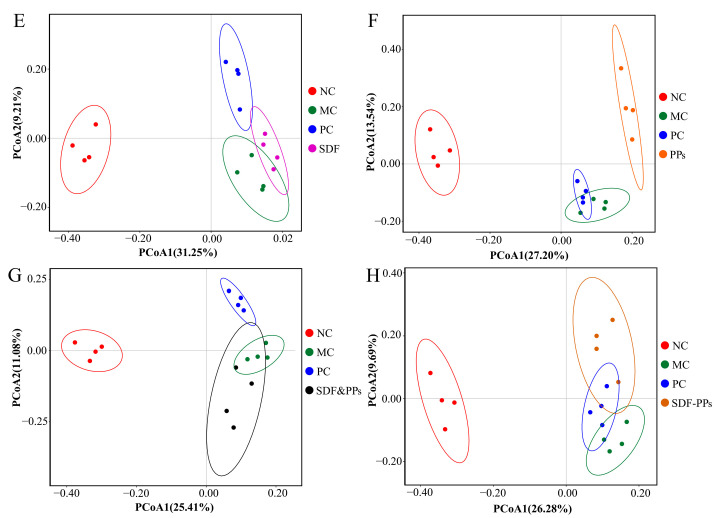
Effect of diet on changes in gut microbiota composition and relative abundance in HFD mice. (**A**) Dilution curves. (**B**) ACE indices. (**C**) Chao1 indices. (**D**) Shannon’s indices. (**E**–**H**) PCoA analysis based on binary Jaccard: (**E**) NC, MC, PC, and SDF groups; (**F**) NC, MC, PC, and PPs groups; (**G**) NC, MC, PC, and SDF&PPs groups; (**H**) NC, MC, PC, and SDF-PPs groups. (a–c) Mean values with different letters in the same figure are significantly different (*p* < 0.05) according to Duncan’s multiple range test. Data are expressed as means ± SDs (n = 4). NC: normal control group; MC: model control group; PC: positive control group (simvastatin 10 mg/kg); SDF: soluble dietary fiber group (250 mg/kg); PPs: polyphenols group (150 mg/kg); SDF&PPs: physical mixing group (250:150 ratio blend); SDF-PPs: composite group (400 mg/kg).

We analyzed the mice’s gut microbiota by 16S rDNA sequencing and detected an average of 56,650 sequences per sample (minimum: 45,846) after clipping de-duplication and chimeric deletion. As can be seen in Figure 4A, the samples’ dilution curves responded to the depth of their sequencing. The curves all flattened out, showing that the sequencing depth was adequate for the subsequent analysis phase.

We also measured the α-diversity of the flora by Chao1, ACE, and Shannon indices, as shown in Figure 4B–D. The intestinal flora of the mice were changed by the high-fat dietary intervention. When compared to the NC group, the MC group’s Chao1, ACE, and Shannon indices were significantly lower, showing that a high-fat diet reduces the variety and abundance of bacterial flora. The data were analyzed for beta diversity to compare the degree of similarity that exists between samples in terms of species diversity. The binary Jaccard technique was used to create PCoA plots, and the closer the samples were to one another on the coordinate plot, the more similar they were. The NC and MC groups were clearly distinguished from one another on the PCoA plots (Figure 4E–H), demonstrating that the mice’s gut flora were considerably affected by the high-fat diet. The SDF-PPs and MC groups seemed to be apart, while the SDF and SDF&PPs groups were closer to the MC group. The findings demonstrated that in hyperlipidemic mice, SDF-PPs restored the diversity and quantity of gut flora. The PPs and MC groups also showed separation, which is noteworthy. 

**Figure 5:**
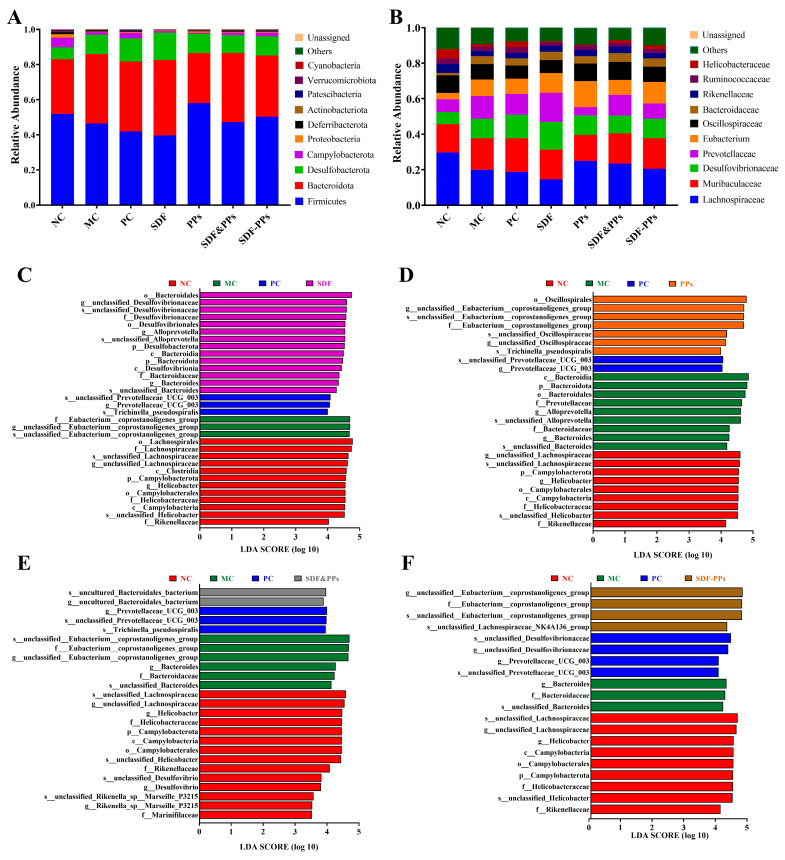
Effect of dietary intervention on intestinal flora species in hyperlipidemic mice. (**A**) Changes at the phylum level in the groups. (**B**) Changes at the genus level in the groups. (**C**–**F**) LEfSe analysis: (**C**) NC, MC, PC, and SDF groups; (**D**) NC, MC, PC, and PPs groups; (**E**) NC, MC, PC, and SDF&PPs groups; (**F**) NC, MC, PC, and SDF-PPs groups. Data are expressed as means ± SDs (n = 4). NC: normal control group; MC: model control group; PC: positive control group (simvastatin 10 mg/kg); SDF: soluble dietary fiber group (250 mg/kg); PPs: polyphenols group (150 mg/kg); SDF&PPs: physical mixing group (250:150 ratio blend); SDF-PPs: composite group (400 mg/kg).

The structuring of the bacterial communities at the phylum level for each group of samples is shown in Figure 5A. In contrast to the NC group, the MC group had higher levels of *Bacteroidota*, which are known to play a key role in the development of hyperlipidemia. When compared to the MC group, *Firmicutes* were increased and *Bacteroidota* were reduced in the SDF-PPs group. In addition, genus-level histograms were analyzed using the top 10 enriched genera and used to identify specific genera altered by the SDF-PPs intervention (Figure 5B). Subsequently, SDF-PPs had a reversal effect on the increase in *Desulfovibrionaceae* and *Prevotella* and increased the content of *Lachnospiraceae*. 

Biomarkers that were statistically different between groups might be identified using linear discriminant analysis (LDA) of effect size (LEfSe). As shown in Figure 5C–F, comparing the four groups analyzed by LEfSe, the NC group had the highest species diversity, whereas a high-fat diet led to a decrease in gut species diversity. In our study, we found that SDF was associated with the enrichment of *c_Bacteroidia* and *c_Desulfovibrionia* (Figure 5C), PPs was associated with the enrichment of *o_Oscillospirales* (Figure 5D), SDF&PPs was associated with the *g_uncultured_Bacteroidales_bacterium* enrichment (Figure 5E), and SDF-PPs was associated with enrichment of *f_Eubacterium_coprostanoligenes_group* and *s_unclassified_Lachnospiraceae_NK4A136_group* (Figure 5F).

## 4. Discussion

In recent years, active components in plants have received more and more attention, and the development of lipid-lowering functional components in plants is a current research hotspot. It was found that the product of the interaction between SDF and PPs had better in vitro cholesterol adsorption and lipid adsorption. Therefore, in this study, we prepared complexes of soluble dietary fiber and polyphenols from lotus root and compared the effects of SDF, PPs, SDF&PPs, and SDF-PPs derived from lotus root in preventing hyperlipidemia.

Hyperlipidemia leads to dyslipidemia, and lipid levels of LDL-C, HDL-C, TC, and TGs, as well as ALT, ALP, and AST enzyme activities, are commonly used as clinical criteria for the diagnosis of hyperlipidemia [13]. Our results showed that SDF-PPs significantly reduced the plasma levels of TC, TGs, and LDL-C and inhibited the plasma activities of ALT, ALP, and AST in HFD-induced mice. This means that SDF-PPs have great potential in preventing hyperlipidemia. In addition, hyperlipidemia is accompanied by obesity, and lipids tend to accumulate in liver cells. In the results for liver H&E staining, it can be seen that HFD-induced mice showed a large number of fat droplets in their liver tissues, and this phenomenon was mitigated after the drug intervention, which further illustrates the beneficial effect of SDF-PPs on hyperlipidemic mice. 

Over the past few years, there has been increasing evidence that dyslipidemia is strongly associated with oxidative stress [13]. Antioxidant enzymes that remove reactive oxygen species in vivo include TAOC, SOD, and GSH-Px. MDA and AGEs are biomarkers of oxidative stress. Oxidative stress is thought to be a major source of cellular damage and an inflammatory response [22]. Additionally, studies on in vitro animals have demonstrated the pro-mercurial effects of inflammatory responses or increased AGEs contents on heightened oxidative stress. The generation of oxidative stress is significantly correlated with the level of AGEs [23]. Polyphenols are also a good dietary source for inhibiting AGEs [24]. In the present study, the SDF-PPs intervention increased hepatic TAOC, SOD, and GSH-Px activities, while it decreased MDA levels and AGEs contents and was superior to the SDF, PPs, and SDF&PPs treatments. This may be related to the interaction between soluble dietary fiber and polyphenols, and previous studies have shown that the antioxidant activity of polyphenols bound to dietary fiber can be regenerated in solution by extractable antioxidants, which provide electrons or hydrogen atoms to the former to regenerate them [9].

Studies have linked chronic, systemic inflammation to unhealthy eating patterns [25]. Inflammation is a protective defense response and the cause of many diseases in the body. A study showed that levels of inflammatory factors are also elevated in the livers of mice with high-fat-diet-induced hyperlipidemia [26]. In this study, the livers of mice in the MC group had lower levels of the anti-inflammatory factor TGF-β and higher levels of the inflammatory factors IL-6, IL-1β, and TNF-α. Compared with the MC group, the SDF, PPs, SDF&PPs, and SDF-PPs groups had lower levels of IL-6, IL-1β, and TNF-α and higher levels of TGF-β (*p* < 0.05). In addition, the effect of SDF-PPs on inflammatory factors was greater than that of SDF, PPs, and their physical mixtures. The combined experimental results showed that SDF-PPs could regulate the inflammatory state of the body, protect against liver injury, and alleviate hyperlipidemia in HFD-induced mice.

To further elucidate the potential mechanisms underlying the regulatory effects of SDF-PPs on lipid metabolism, we investigated changes in genes and proteins associated with the AMPK signaling pathway. AMPK is a well-known energy sensor and metabolic regulator that can inhibit fatty acid synthesis by inhibiting anabolism or promoting catabolism to promote fatty acid oxidation. Phosphorylated AMPK (p-AMPK) inhibits lipid synthesis by phosphorylating acetyl coenzyme A carboxylase 1 (ACC1) and decreasing the activity of ACC1, thereby decreasing substrate supply to fatty acid synthase. 3-hydroxy-3-methylglutaryl-coenzyme A reductase, often known as HMG-CoA, is the enzyme that controls the rate at which cholesterol is produced and is similarly controlled by AMPK. When HMG-CoA is phosphorylated, its activity is diminished, which in turn reduces the production of cholesterol [27,28]. Recent years have seen a surge in interest in research on AMPK regulation of lipid metabolism. It has been hypothesized that sea buckthorn fruit oil extract can regulate body weight and fat growth in golden hamsters by altering the expression of key genes in the AMPK and Akt pathways and encouraging the phosphorylation of AMPK and Akt proteins [29]. By controlling AMPK activity and lipid-metabolizing proteins, *Ulmus Macrocarpa Hance* extract inhibited the buildup of lipids. Additionally, a similar pattern of gene expression levels was validated in HepG2 cells treated with oleic acid (OA) [11]. The SDF-PPs intervention effectively reversed the reduction in p-AMPK/AMPK, down-regulated levels of FAS and HMG-CoA proteins, and up-regulated levels of CPT1 proteins. It is worth noting that SDF and PPs can also to some extent reverse the reduction in p-AMPK/AMPK, but SDF&PPs showed poor results, though the specific reasons for this need to be further studied. Additionally, the expression of genes involved in the production of fat is controlled by AMPK. Sterol regulatory element-binding protein-1 (SREBP-1) is expressed less when AMPK is activated by phosphorylation. The lipogenesis-regulating transcription factor SREBP-1 also regulates the expression of the ABCA1, FAS, LXRa, and ACC1 genes [30,31]. This study found that significantly higher levels of SREBP-1 and ACC1 were expressed in response to a high-fat diet, with significantly lower expression of LXRa and ABCA1, compared to the NC group. While all four administration modes improved this phenomenon, SDF-PPs showed the optimal effect. It is noteworthy that the PPs group also showed good results in terms of inhibiting SREBP-1 and elevating LXRa expression, which did not differ much from the improvement effect of SDF-PPs. In summary, SDF-PPs inhibit fat accumulation in the liver by phosphorylating AMPK, down-regulating FAS and HMG-CoA, up-regulating CPT1 expression, and inhibiting the activity of the transcription factor SREBP-1 and its regulated adipogenic genes.

Since the gut microbiota can affect satiety, liver cholesterol metabolism, muscle lipid oxidation, adipose tissue energy storage, and gut barrier integrity, it can control host lipid metabolism and contribute to the development of hyperlipidemia [32]. Numerous plant species have shown therapeutic potential to treat hyperlipidemia by modifying the gut microbiota, and targeted therapies based on the gut microbiota have so far improved hyperlipidemia with satisfactory results [33,34]. In our study, it was found that *Firmicutes* and *Bacteroidota* were the major components in each group. Han et al. showed that a high-fat diet resulted in a higher abundance of *Firmicutes* and *Bacteroidota* [12]. The two most prevalent prokaryotes in humans are *Firmicutes* and *Bacteroidota*, and the ratio of *Firmicutes* to *Bacteroidota* (F/B) is widely recognized as having an important influence on the maintenance of normal intestinal homeostasis [35]. In this study, the F/B ratio was 1.67 in the NC group and 1.17 in the MC group, and the F/B ratio was reduced in high-fat mice, whereas the SDF-PPs intervention normalized F/B ratios by increasing the number of *Firmicutes* while decreasing the number of *Bacteroidota*. According to earlier research, this is accurate [15]. When compared to the NC group, the intestinal tracts of the mice given a high-fat diet had fewer *Lachnospiraceae* and more *Desulfovibrionaceae* and *Prevotellaceae*, which is consistent with studies in the literature [17,34]. *Lachnospiraceae* are involved in carbohydrate metabolism, fermenting acetic and butyric acids to provide energy for the host [36,37]. Inflammatory disorders had a positive correlation with *prevotellaceae* [38], and *Desulfovibrionaceae* belongs to the genus of harmful intestinal Bacteria, which was positively associated with the indicators of hyperlipidemia in the body [39]. Therefore, it can be speculated that dyslipidemia in the sera of high-fat mice may be related to altered microbial structure. Finally, we found that SDF-PPs were associated with the enrichment of *s_unclassified_Lachnospiraceae_NK4A136_group* by LEfSe analysis. It was shown that *s_unclassified_Lachnospiraceae_NK4A136_group* is a probiotic considered as a potential target for alleviating obesity, hyperlipidemia and metabolic syndrome [40]. By modifying the gut microbiota, these microbial alterations imply that SDF-PPs may reduce hyperlipidemia and its associated complications in HFD mice. However, since SDF-PPs are plant extracts containing a variety of phytochemicals, it is necessary to identify the components of SDF-PPs that exert hyperlipidemia-relieving effects, and since lipid metabolism in vivo is a complex, multi-targeted process, the specific mechanism by which SDF-PPs relieve hyperlipidemia needs to be further investigated.

## 5. Conclusions

In the present study, we showed favorable lipid-lowering effects of SDF-PPs in HFD-induced obese mice. The results show that the lipid-lowering effect of SDF-PPs might be related to the activation of the AMPK pathway, which was mainly manifested by phosphorylation of AMPK, down-regulation of FAS and HMG-CoA, up-regulation of the expression of CPT1, and down-regulation of the expression of the transcription factor SREBP-1 and the liposynthesis genes, which resulted in a reduction in the production of fat and cholesterol in the liver. In addition, SDF-PPs improved the structure of gut microbiota and increased the proportion of *Firmicutes* and *Bacteroidota* in the guts of HFD mice. Taking these results together, we can conclude that SDF-PPs can be used as a promising dietary supplement for the prevention of hyperlipidemia. This study provides a basis for the development of novel dietary therapies for the treatment of hyperlipidemia.

## Figures and Tables

**Table 1:** Effects of different dietary interventions on the weight gain, food intake, and organ indices of mice.

	NC	MC	PC	SDF	PPs	SDF&PPs	SDF-PPs
Starting weight (g)	17.02 ± 1.47 a	17.18 ± 0.56 a	17.21 ± 0.78 a	17.11 ± 1.45 a	17.16 ± 0.89 a	17.10 ± 1.21 a	17.28 ± 0.57 a
Final weight (g)	20.02 ± 0.99 c	22.11 ± 0.28 a	20.48 ± 1.00 bc	21.26 ± 0.95 bc	21.15 ± 1.17 ab	20.94 ± 0.63 bc	20.77 ± 0.99 ab
Weight gain (g)	3.00 ±0.83 c	4.93 ± 0.71 a	3.26 ± 0.86 bc	4.14 ± 0.85 bc	3.99 ± 0.99 ab	3.84 ± 0.83 bc	3.49 ± 0.67 ab
Food intake (g/d)	15.31 ± 1.32 a	13.76 ± 0.90 b	13.93 ± 0.73 b	13.61 ± 1.49 b	13.10 ± 2.22 b	13.63 ± 1.31 b	13.96 ± 1.62 b
Energy intake (Kcal/d/g)	46.59 ± 4.02 a	54.23 ± 3.56 b	54.90 ± 2.88 b	53.70 ± 5.17 b	53.65 ± 5.85 b	55.02 ± 6.39 b	51.61 ± 8.76 b
Liver index (%)	3.30 ± 0.19 c	4.09 ± 0.19 a	3.97 ± 0.25 ab	3.83 ± 0.18 b	3.79 ± 0.27 ab	3.71 ± 0.26 c	3.61 ± 0.29 b

(a–c) Mean values with different letters in the same row are significantly different (*p* < 0.05) according to Duncan’s multiple range test.

**Table 2:** Effects on enzyme activity and inflammation indicators in different groups.

	NC	MC	PC	SDF	PPs	SDF&PPs	SDF-PPs
Enzyme Activity							
TAOC (mM)	0.46 ± 0.03 a	0.26 ± 0.05 d	0.38 ± 0.04 b	0.29 ± 0.03 cd	0.36 ± 0.04 b	0.34 ± 0.03 bc	0.44 ± 0.04 a
GSH-Px (U/mgprot)	49.52 ± 4.95 a	34.38 ± 1.72 c	43.10 ± 2.90 b	34.55 ± 1.26 c	37.68 ± 2.74 c	37.56 ± 2.30 c	43.51 ± 2.80 b
MDA (nmol/mgprot)	2.96 ± 0.51 d	7.26 ± 0.33 a	4.65 ± 0.48 bc	4.12 ± 0.23 c	4.14 ± 0.56 c	5.05 ± 0.44 b	3.24 ± 0.67 d
SOD (U/mgprot)	9.52 ± 0.54 a	7.03 ± 0.34 c	8.52 ± 0.28 b	6.59 ± 0.53 c	7.15 ± 0.22 c	6.70 ± 0.23 c	8.04 ± 0.37 b
AGEs (pg/g)	2052.70 ± 72.32 e	4125.66 ± 89.95 a	2319.23 ± 139.53 d	3634.31 ± 148.72 b	3577.50 ± 158.84 b	3469.02 ± 219.11 b	3211.87 ± 83.15 c
Inflammation Indicators							
IL-6 (pg/mg)	426.06 ± 20.93 d	1184.50 ± 68.42 a	471.68 ± 34.60 d	808.31 ± 72.31 b	682.77 ± 85.36 c	652.48 ± 40.11 c	587.58 ± 74.46 c
IL-1β (pg/mg)	390.03 ± 15.04 e	1018.36 ± 73.31 a	417.75 ± 42.04 e	727.49 ± 30.29 b	529.49 ± 92.99 cd	544.45 ± 28.64 c	455.84 ± 20.80 de
TNF-α (pg/mg)	2610.47 ± 123.48 c	6626.71 ± 230.65 a	2828.00 ± 281.35 c	3805.20 ± 277.90 b	3877.26 ± 580.97 b	3812.87 ± 182.58 b	3387.50 ± 366.31 b
TGF-β (pg/mg)	6610.59 ± 251.67 a	2559.06 ± 201.59 f	5327.29 ± 103.40 b	3875.05 ± 186.15 c	4511.18 ± 149.53 d	4556.29 ± 252.70 cd	4827.86 ± 155.42 c

(a–f) Mean values with different letters in the same row are significantly different (*p* < 0.05) according to Duncan’s multiple range test.

## Data Availability

Data will be made available on request.

## Supplementary Materials

The following supporting information can be downloaded at: https://www.mdpi.com/article/10.3390/antiox13040466/s1. Figure S1. The animal experimental design; normal control group (NC); model control group (MC); positive control group (simvastatin 10 mg/kg, PC); soluble dietary fiber group (250 mg/kg, SDF); polyphenols group (150 mg/kg, PPs); physically mixing group (250:150 ratio of blended, SDF&PPs); composite group (400 mg/kg, SDF-PPs). Table S1. Primer sequence.
