# Supplementary material for: Complexes of Soluble Dietary Fiber and Polyphenols from Lotus Root Regulate High-Fat Diet-Induced Hyperlipidemia in Mice
Source: Antioxidants (Basel). 2024 Apr 16;13(4):466. doi: 10.3390/antiox13040466 (PMC11047371; doi:10.3390/antiox13040466)
Supplement: Supplementary file 1 [file antioxidants-13-00466-s001.zip › antioxidants-2904775-supplementary.pdf]

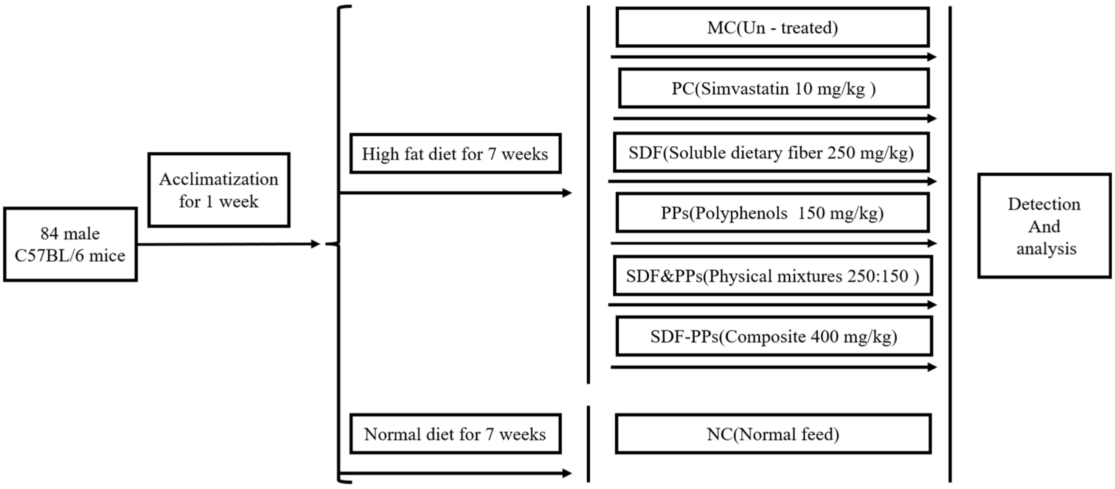

Figure S1. The animal experimental design; normal control group (NC); model control group (MC); positive control group (simvastatin 10 mg/kg, PC); soluble dietary fiber group (250 mg/kg, SDF); polyphenols group (150 mg/kg, PPs); physically mixing group (250:150 ratio of blended, SDF&PPs); composite group (400 mg/kg, SDF-PPs).

Table S1. Primer sequence.

Gene	Primer	Sequence (5'-3')	PCR Products	NCBI Accession No.
MusGAPDH	Forward	ATGGGTGTGAACCACGAGA	229bp	NM_001289726.2
	Reverse	CAGGGATGATGTTCTGGGCA		
MusHGM-COA	Forward	GAGATCATGTGCTGCTTCGG	194bp	NM_001360165.1
	Reverse	CTTTGGGTTACGGGGTTTGG		
MusABCA1	Forward	CAATGATTTGATGAAGAACTTGGAG	106bp	NM_0.13454.3
	Reverse	TGGTGTGTCAGGTGTATAGAGA		
MusACC1	Forward	ACCGTCTGCTGGGAAGTTAA	179bp	NM_001198214.2
	Reverse	CTATCACACAGCCAGGGTCA		
MusFAS	Forward	GTCCTGCCTCTGGTGCTT	168bp	NM_001146708.1
	Reverse	TTCAGGTTGGCATGGTTGAC		
MusLXRa	Forward	CACTACCCACTCTAACG	107bp	NM_001177730.1
	Reverse	TCTTCCCTTGACTCCT		
MusSREBP-1	Forward	GCAGTGGTGGTAGTGACTCT	152bp	NM_001313979.1
	Reverse	AGGATTGCAGGTCAGACACA		
